# Genomic Characterization of SARS-CoV-2 Variants from Clinical Isolates during the COVID-19 Epidemic in Mauritania

**DOI:** 10.3390/genes15030361

**Published:** 2024-03-14

**Authors:** Jemila Deida, Nasserdine Papa Mze, Mamadou Beye, Sidi Mohamed Ahmed, Ahmed El Bara, Mohamed Abdallahi Bollahi, Leonardo Basco, Ali Ould Mohamed Salem Boukhary, Pierre-Edouard Fournier

**Affiliations:** 1 UR-Génomes et Milieux, Université de Nouakchott, Nouakchott BP 880, Mauritania; jemilasidiali@gmail.com (J.D.); alimedsalem@gmail.com (A.O.M.S.B.); 2 Institut National de Recherche en Santé Publique, Nouakchott BP 695, Mauritania; sm.ahmed927@gmail.com (S.M.A.); elbaraahmed@yahoo.fr (A.E.B.); bollahi@yahoo.com (M.A.B.); 3 Aix Marseille University, AP-HM, SSA, VITROME, 13005 Marseille, France; npapamze@gmail.com (N.P.M.); bemamadou@gmail.com (M.B.); lkbasco@yahoo.fr (L.B.); 4 IHU-Méditerranée Infection, 13005 Marseille, France

**Keywords:** coronavirus, COVID-19, Mauritania, SARS-CoV-2, variants

## Abstract

The rapid genetic evolution of the severe acute respiratory syndrome coronavirus 2 (SARS-CoV-2) during the coronavirus disease 2019 (COVID-19) pandemic has greatly challenged public health authorities worldwide, including in Mauritania. Despite the presence of the virus in Mauritania, only one study described its genomic variation during the course of the epidemic. The purpose of the present study was to document the genomic pattern of SARS-CoV-2 variants from clinical isolates during the COVID-19 outbreak in Mauritania, from September to November 2021. The whole genomes from 54 SARS-CoV-2 strains detected in nasopharyngeal swabs with a cycle threshold value ≤ 30 were successfully sequenced using next-generation sequencing (NGS) and the Illumina protocol. The mean genome coverage (±standard deviation) was 96.8% (±3.7). The most commonly identified clade was 21J (57.4%), followed by 21D (16.7%), 20A (11.1%), and 20B (9.2%). At the level of lineages, the majority of the samples were Delta variants with the sub-lineage AY.34 (or B.1.617.2.34). Among the 54 SARS-CoV-2 isolates that were successfully sequenced, 33 (61.1%) came from vaccinated individuals, and 21 (38.9%) were from unvaccinated individuals. Several SARS-CoV-2 variants were present in Mauritania between September and November 2021. As Mauritania, like many West African countries, is resource-limited regarding viral genome sequencing facilities, establishment of mutualized sub-regional sequencing platforms will be necessary to ensure continuous monitoring of mutations in viral genomes and track potential reduction in COVID-19 vaccine efficacy, increased transmissibility, and disease severity.

## 1. Introduction

Coronavirus disease 2019 (COVID-19) is an acute respiratory tract infection due to the emerging coronavirus called severe acute respiratory syndrome coronavirus 2 (SARS-CoV-2), a single-stranded RNA virus of positive polarity. COVID-19 was first reported in December 2019 in the city of Wuhan, Hubei Province, China, in patients with pneumonia. From there, it spread quickly across China and then to the rest of the world [1]. Over three years (i.e., from 20 January 2020 to 5 May 2023), COVID-19 was considered by the World Health Organization (WHO) as a public health emergency of global scope [2]. As of 17 December 2023, over 772 million confirmed cases and nearly seven million deaths have been reported globally [3]. Africa represents 1.2% of positive cases and 2.5% of deaths worldwide [3]. 

According to the WHO, 65% of Africans were infected with SARS-CoV-2 between 2020 and 2021 [4]. According to the same source, exposure to SARS-CoV-2 in Africa rose sharply from 3% (with a margin of error ranging from 1% to 9.2%) in June 2020 to 65% (margin of error between 56.3% and 73%) in September 2021, equivalent to 800 million infections, while only 8.2 million cases were diagnosed and officially reported over the same period. The level of exposure to the virus has risen sharply following the appearance of the Beta and Delta variants. Seroprevalence varied widely within and between countries in Africa. Seroprevalence was higher in more densely populated urban centers than in rural areas. Seroprevalence also varied between age groups, with children aged 0–9 years old being less infected than adults. Exposure to the virus also differed from one sub-region to another on the continent, being higher in East, West, and Central Africa. In addition, Africa has reported a fewer number of severe cases compared to other regions of the world. One of the major factors underlying this trend is likely to be the relatively high proportion of children and young people in African countries although other possible protective factors, such as genetics and environment, may also be involved [5]. 

The genome of SARS-CoV-2 is undergoing rapid evolution that has given rise to several variants as the result of mutations. For instance, more than 25,000 mutations have already been reported in the SARS-CoV-2 genome since the emergence of the virus in the human populations [6]. Among these mutations, those that occur in the gene encoding the Spike (S) glycoprotein are the most important for viral transmission and pathogenicity because it serves as a ligand and is essential for virus entry through interaction with the human angiotensin-converting enzyme 2 (hACE2) receptor on the host cells [7]. More than 4700 distinct mutations in the S protein gene were reported during the first two years of the pandemic [8]. Since its emergence in 2020, numerous virus variants at the origin of different COVID-19 epidemic waves have been identified, including Alpha (B.1.1.7, first reported in the United Kingdom), Delta (B.1.617.2, first reported from India), and Omicron (B.1.1.529, first reported from South Africa) variants characterized by their high level of human-to-human transmissibility, disease severity, and immune escape [9,10,11,12]. For instance, the Omicron variant was shown to spread easily, even among vaccinated populations [13]. 

In the West African country of Mauritania, the first COVID-19 case was detected on 13 March 2020 in a 40-year-old Australian expatriate traveling from abroad [14]. Since that time, the country experienced successive epidemic waves affecting all regions characterized by the emergence of variants of concern detected as early as in September 2020 [15,16,17]. As of 19 December 2023, there have been a total of 63,764 confirmed cases and 997 (1.56%) deaths due to COVID-19 in Mauritania [18].

Although COVID-19 is present in Mauritania, the limited number of studies conducted on the disease were mainly focused on its epidemiological characteristics [14,15] and the capacities of the local healthcare system to cope with the pandemic [19]. Studies addressing the dynamic of the SARS-CoV-2 variants circulating in the country during the course of the epidemic are scarce [16,17]. The objective of this study was to determine the genomic characteristics of SARS-CoV-2 clinical isolates during an episode of the COVID-19 epidemic in Mauritania.

## 2. Materials and Methods

### 2.1. Study Site

Mauritania is a country in West Africa, located between 15 and 27 degrees north latitude and 5 and 17 degrees west longitude. It is bordered to the north by Western Sahara and Algeria; to the east by Mali; and to the south by Mali and Senegal. To the west, it borders the North Atlantic Ocean with a 600 km coastline, stretching from Ndiago in the south to Nouadhibou in the north. It has a surface area of 1,030,700 km^2^ and a population of around 4,500,000 [20].

### 2.2. Sample Collection

Mauritania was struck by the third wave of COVID-19 epidemic in the second half of 2021. Due to inadequate laboratory facilities in many health centers and hospitals, samples from symptomatic patients suspected to be COVID-19-positive were collected and sent to the Mauritanian National Institute of Public Health Research (“Institut National de Recherche en Santé Publique”, INRSP), where virological diagnosis was centralized and undertaken by reverse transcription-polymerase chain reaction (RT-PCR). Among the available samples during this epidemic, a total of 103 positive nasopharyngeal swab samples collected from September to November 2021 were selected by simple random sampling from the banked nasopharyngeal samples at the INRSP. Socio-demographic and clinical information of individuals from whom nasopharyngeal swabs were collected was also obtained from the INRSP patient records. All patient data were anonymized. The present study was reviewed and approved by the Ethics Committee of the University of Nouakchott under reference no. 2020-010. 

### 2.3. Viral RNA Extraction and Ampslification

Viral RNA was extracted from nasopharyngeal samples using the MagMax Viral/Pathogen nucleic acid isolation kit (Thermo Fisher Scientific, Waltham, MA, USA) according to manufacturer’s instructions. The quality and concentrations of all RNA extracts were measured with a NanoDrop spectrophotometer (Thermo Fisher Scientific). Extracted viral RNAs were immediately used for viral detection and, if positive, sequenced or stored at −20 °C until use. 

Real-time RT-PCR was performed to detect SARS-CoV-2 RNA using a commercial kit for real-time fluorescent RT-PCR for the detection of SARS-CoV-2 (BGI Health (HK) Co. Ltd., Shenzhen, China) following the manufacturer’s instructions. The BGI kit detects SARS-CoV-2 in a one-step strategy using the ORF1ab probe. Quantitative real-time RT-PCR reaction was performed using a LightCycler^®^ 480 Instrument II (Roche, Basel, Switzerland). RT-PCR was performed with a single thermal cycling program using 30 µL of mix (containing the enzyme and primers) and 10 µL of eluted RNA. The RT-PCR program consisted of an initial step at 50 °C for 20 min and denaturation step at 94 °C for 10 min, followed by 40 cycles at 95 °C for 15 s and 60 °C for 30 s. Only positive samples with cycle threshold (Ct) values ≤ 30 were retained for sequencing.

### 2.4. SARS-CoV-2 Whole Genome Sequencing

Libraries were prepared following the Illumina COVIDSeq protocol (Illumina Inc., San Diego, CA, USA) as previously described [21]. This protocol comprises four steps: cDNA preparation, target amplification, library preparation and pooling, and sequencing. First, the first strand of cDNA was synthesized using reverse transcriptase and random hexamer primer. In this step, the extracted RNA (8.5 µL) was first annealed using the elution prime fragment 3HC (8.5 µL) and then transcribed into cDNA using reverse transcriptase HT (1 µL). 

In the second step, the synthesized cDNA was amplified by performing two multiplex PCR amplifications using two non-overlapping COVIDSeq primer pools 1 and 2 (CPP1 and CPP2, respectively). The sequences of these specific primer pools (ARTIC v3 SARS-CoV-2 primer sets) were retrieved from an open source platform [22]. The thermal cycler was programmed as follows: after the initial denaturation step (98 °C for 3 min), 35 cycles of denaturation (98 °C for 30 s) and annealing/extension (65 °C). 

In the third step, the PCR products were tagged using 4 µL of Enrichment BLT HT beads for library preparation, followed by adapter ligation with 10 µL of the Illumina IDT^®^ PCR indexes sets 1, 2, 3, and 4. The labeled PCR products were amplified by another PCR amplification: initialization (72 °C for 3 min), initial denaturation (98 °C for 3 min), seven cycles of amplification (denaturation at 98 °C for 20 s, hydridization at 60 °C for 30 s, and extension at 72 °C for 1 min), and the final extension (72 °C for 3 min). Each 96-well PCR plate included a positive control (COVIDSeq HT, CPC HT) and a negative control. An individual library was derived from 96 PCR-amplified products in a 96-well PCR plate; 5 µL of each PCR-amplified product were pooled and mixed in a 1.5-mL microfuge tube to constitute an individual library. Individual libraries were quantified using the Qubit 2.0 fluorometer (Invitrogen, Villebon-sur-Yvette, France) and pooled in equimolar concentration as recommended by Illumina. The pooled PCR products were diluted (1:10) in the resuspension buffer, and the concentration was adjusted to 4 nM. 

In the fourth and last step, the normalized library pools (25 µL of each pooled samples adjusted to the concentration of 4 nM; volume of the final library, 2.25 µL) were diluted to a final concentration of 0.5 nM, as recommended by Illumina, denatured, neutralized (mixture of 4 µL of 0.2 N NaOH and 5 µL of 400 mM of Tris-HCl), and mixed with 63 µL of ExAmp mix for sequencing using Nova Seq system (Illumina Inc.).

### 2.5. Data Analysis

Dragen Bcl Convert v3.9.3 and FreeBayes v1.3.5 pipelines were used to process base and variant callings, respectively [23,24]. The bwa-mem2 tool v2.2.1 was used for mapping, based on the genome of Wuhan-Hu-1 isolate (GenBank accession no. NC_045512.2) [25,26]. The resulting sequence was cleaned using SAMtools program v1.13 [27,28]. Consensus genomes were built with the BCFtools program v1.13 [29]. 

All nucleotide and deduced amino acid sequences were aligned against the Wuhan-Hu-1 isolate genome as reference, and sequence modifications were detected using the Nextclade tool [30,31,32]. Phylogenetic clades and lineages were assigned using the Nextclade web application [30,31,32] and Pangolin tool [33,34], respectively. Phylogenetic trees were obtained using Nextstrain [30]. 

Qualitative data were grouped and analyzed using Fisher’s exact test. MedCalc version 22.021, an open source software [35], was used to calculate the *p*-values. A two-tailed *p*-value < 0.05 was considered statistically significant. 

## 3. Results

### 3.1. Characteristics of Clinical Isolates

In this study, 103 nasopharyngeal swab samples tested positive by quantitative RT-PCR in the virology laboratory of the INRSP were transferred to the Institut Hospitalo-Universitaire—Méditerranée Infection (IHU-MI; Marseille, France) for whole genome sequencing. Of these samples, 81 (78.6%) were confirmed to be positive by quantitative RT-PCR. Of these 81 confirmed SARS-CoV-2 samples, 54 (66.7%) with Ct values ranging from 12 and 30 were successfully sequenced.

### 3.2. Patient Characteristics

The great majority of the patients whose viral samples were successfully sequenced (n = 54) were men (n = 46; 85.2%); eight were women (14.8%). Their mean (±standard deviation) and median ages were 36.8 (±13.6) and 40 years, respectively. Among 54 SARS-CoV-2 isolates that were sequenced, 21 (38.9%) were sampled from unvaccinated individuals, and 33 (61.1%) were from vaccinated individuals. The vaccinated individuals included 17 (51.5%) patients who received the Sinopharm COVID-19 vaccine (Beijing Bio-Institute of Biological Products), 14 (42.4%) who were vaccinated with the first dose of Vaxzevria vaccine (Oxford/AstraZeneca, Cambridge, UK), and 2 (6.1%) who were vaccinated with the Janssen COVID-19 vaccine (Janssen Pharmaceutical Co./Johnson & Johnson, Titusville, NJ, USA). The INRSP patient records indicated that 26 of 54 (48%) sequenced samples were from hospitalized patients, while the others (n = 28; 52%) were from outpatients with no history of travel and classified as community transmission. Minimal clinical data were available in the INRSP patient records. The patients’ original hospital records and outpatient registry records were not available for consultation.

### 3.3. SARS-CoV-2 Genome Sequence Analysis and Variants

SARS-CoV-2 clinical isolates were sequenced using the Illumina protocol. High-resolution genotyping results were obtained. All samples had a genome sequence coverage between 78.8% and 99.3%, with a mean of 96.8% and a mean depth of 1767.7 x (range, 1049 to 2610) (Table S1: Genomic features of Mauritanian clinical isolates of SARS-CoV-2). The genomic sequences were submitted to GenBank (accession numbers OR353179–OR353230).

Of 54 SARS-CoV-2 isolates, 36 (66.9%) were variants of concern (VOC), including 33 (61.2%) Delta variant, 1 (1.9%) Alpha variant, 1 (1.9%) 20A variant, and 1 (1.9%) 20B variant (B.1.1.528) (Table 1). In addition, 15 (27.8%) were variants of interest (VOI) (11 [20.4%] Eta and 4 [7.4%] 20B), while 3 (5.5%) were variants under monitoring (VUM) (20A). Overall, there were 10 different lineages belonging to 6 different clades with AY.34 (or B.1.617.2.34) being predominant (31/54; 57.4%), followed by B.1.525 (11/54; 20.4%), B.1.620 (3/54; 5.5%), and B.1.1.318 (3/54; 5.5%). For six additional variants, only a single isolate was found in the present study. Clade 19A, which was predominant during the first COVID-19 wave, was not detected among the sequenced SARS-CoV-2 isolates. The Delta (21J) variant was identified in 7 of 8 (87.5%) women. It was also predominant in males (26/46; 56.5%). Data analysis did not show any statistically significant association (*p* > 0.05) between the virus variants or clades and the vaccination status of the patients (Table 2).

### 3.4. Characteristic Mutations in the Spike Protein of Delta and Eta Variants

The NGS allowed the analysis of mutations and their deduced amino acid substitutions of all SARS-CoV-2 variants (Table S1: Genomic features of Mauritanian clinical isolates of SARS-CoV-2). As the most notable mutations that are suspected to affect transmissibility and pathogenicity of SARS-CoV-2 variants are those found in the spike glycoprotein (S protein), only variations observed in the deduced amino acid sequence of the S protein of the two predominant Delta and Eta variants in our samples were analyzed. The results of this analysis are summarized in Table 3.

Comparison with the Wuhan-Hu-1 reference strain genome showed that 29 of 33 Delta variant isolates harbored the characteristic S protein mutations T19R, L452R, T478K, D614G, P681R, and D950N (Table 2). One isolate (MAURCOVID-024) did not have the amino acid substitutions T19R and T478K, and three isolates (MAURCOVID-015, MAURCOVID-047, and MAURCOVID-018) did not have the mutation at position 452. Regarding the Eta variant, all defining single nucleotide polymorphisms (E484K, F888L, Q52R, and Q677H) in the S protein of this variant were observed among the Mauritanian SARS-CoV-2 isolates, except for the isolate MAURCOVID-016 in which both E484K and F888L amino acid substitutions were not present. It is worth noting that all Delta and Eta variant isolates harbored the D614G amino acid substitution in their respective S protein.

### 3.5. Phylogeny of SARS-CoV-2 Isolates

**Figure 1:**
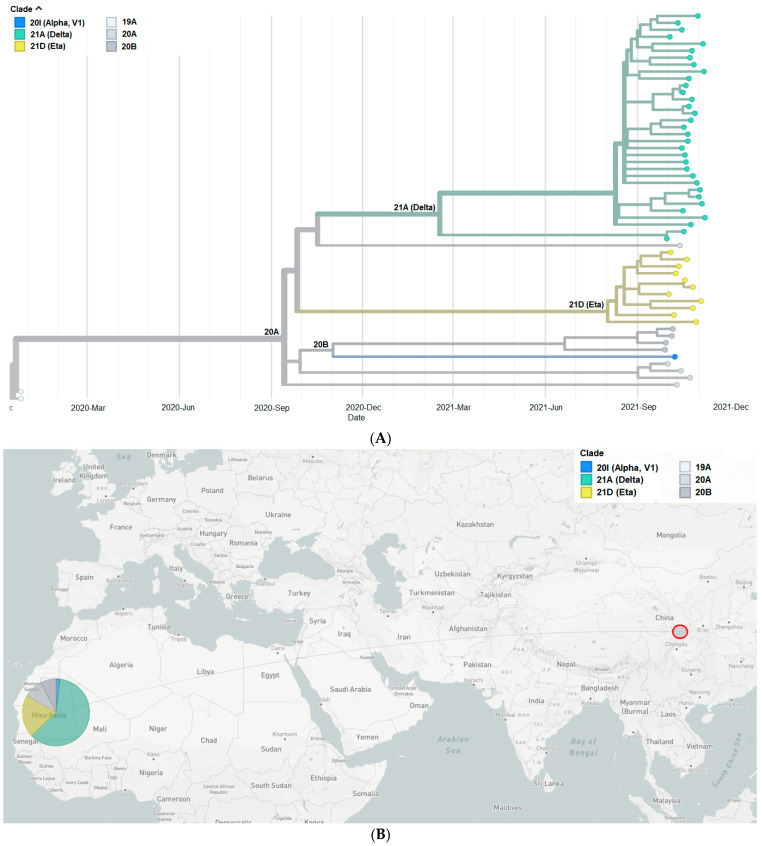
(**A**) Phylogenetic tree constructed based on the dates of emergence of different SARS-CoV-2 variants. (**B**) Map showing China, where the first SARS-CoV-2 case was detected, and Mauritania, the study site. The pie chart indicates the proportion of SARS-CoV-2 clades among the Mauritanian samples.

**Figure 2:**
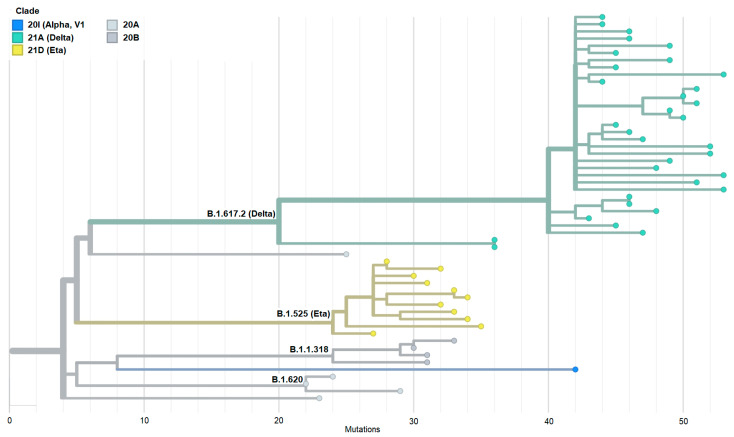
Phylogenetic tree constructed based on the emergence of SARS-CoV-2 variants with different mutations. This figure shows that it is the Delta variant that is in the majority with the highest number of mutations.

To better understand the genetic relationship among 54 Mauritanian SARS-CoV-2 clinical isolates, the phylogenetic tree of these clinical isolates was constructed based on date (Figure 1A,B) and mutation (Figure 2). The 54 sequences obtained in the present study were grouped into six distinct clades indicating the genetic divergence among SARS-CoV-2 isolates and therefore, possible distinct introduction of the virus in the country.

## 4. Discussion

SARS-CoV-2, the etiologic agent of the COVID-19 pandemic, is the most devastating coronavirus of the 21st century to date. Within three years, this respiratory illness caused millions of deaths and enormous economic loss worldwide. The SARS-CoV-2 detection strategy in Mauritania has initially been based on serological tests (i.e., rapid antigen tests). But, very rapidly, as in most countries in the world, a PCR-based approach was introduced as gold standard to accurately detect viral RNA [14]. However, following the emergence of SARS-CoV-2 variants of concern associated with severe clinical signs and symptoms, whole genome sequencing using NGS technique has become the method of choice to timely detect the emergence and spread of new variants with novel mutations and understand the transmission dynamics of the virus worldwide, including in Mauritania [16,17,41]. To document COVID-19 dynamics in Mauritania, a set of SARS-CoV-2 clinical isolates collected from Mauritanian COVID-19-positive patients were analyzed for their genomic profile during the period from September to November 2021, corresponding to the third wave of the COVID-19 epidemic in most West African countries [42]. Starting from 103 quantitative RT-PCR positive nasopharyngeal samples, diagnosed at the INRSP, which was the reference center during the pandemic for SARS-CoV-2 testing in Mauritania, the positivity of 83 of 103 samples was confirmed at the IHU–Méditerranée Infection in Marseille, France. Of 83 samples, 54 with a Ct value ≤ 30 were successfully sequenced. The decrease in the number of positive samples when re-tested at IHU–Méditerranée Infection may have resulted from the degradation of viral RNA during storage and transportation.

Among the Mauritanian SARS-CoV-2 isolates characterized in the present study, the Delta variant (lineage B.1.617.2) predominated, accounting for 61.2% of sequenced genomes (Figure 1A). Similar findings were reported by Abdelmalick et al. in SARS-CoV-2 strains isolated from 13 PCR-positive symptomatic patients sampled five months before our sampling (i.e., 3 March 2021 to 31 May 2021) [17]. However, comparison with data from other West African countries shows that the predominant lineage in the third epidemic wave in West Africa was sub-lineage B.1.617.2.36 (45% of all sequences) and that lineage B.1.617.2 only represented 6% of the collected samples [42].

The Delta variant was first detected in India on 5 October 2020. By 22 November 2021, it had spread to over 179 countries [43]. The Delta variant is among five VOCs designated by the WHO. This variant marked the third wave of the COVID-19 pandemic by its remarkable efficacy in transmission and its enhanced capacity for immune escape [44]. Several characteristic mutations are suspected to allow the Delta variant to be one of the most transmissible variants, of which the most notable gene mutations are those found in the S proteins. The mutations in S protein gene found in the present study in the Mauritanian Delta variant isolates were T19R, L452R, T478K, D614G, P681R, and D950N [45]. The viral S protein is necessary for attachment to the surface of the host cells to enable entry into the cells. The S protein is also the protein that is targeted by the host immune system for eradication of the virus [45].

The second most abundant variant among the sequenced SARS-CoV-2 isolates was the Eta variant and its lineage B.1.525, found in 16.6% of the isolates. This variant was first detected in Nigeria (West Africa). During the same period, this lineage was reported in Mali in 30% of 162 SARS-CoV-2 isolates [46]. It is worth noting that during the third wave of COVID-19 in Mali, the Eta variant was predominant from March to May 2021; then the relative proportion of Delta variant increased from June 2021 to October 2021, which corresponds to our study period. In Senegal, the Eta variant was also present but at a lower proportion (5.26%) [47]. The presence of the Eta variant in Mauritania at such a relatively high proportion is not surprising regarding the geographical proximity with Mali and Senegal and the porosity of the borders despite the measures of travel restriction applied during the pandemic. Spike mutations that are commonly reported in Eta variant include Q52R, E484K, D614G, Q677H, and F888L, of which E484K and F888L in the S2 domain of the S protein are characteristics of the variant [48]. All these mutations were found in the Mauritanian isolates. Some of these mutations have been reported to help the virus to resist the neutralizing antibodies response (E484K) or alter virus transmissibility (Q677H) in the earlier SARS-CoV-2 strains [40]. 

Two other variants, namely 20A (lineage B.1.620) and 20B (lineage B.1.1.318), were also detected among the sequenced SARS-CoV-2 isolates in this study, each with a frequency of 11.1%. The lineage B.1.629 originated from central Africa, most likely from Cameroon [49], while the lineage B.1.1.318 has multiple origins as it was reported from Europe and parts of Africa [50].

Furthermore, our phylogenetic analysis perfectly illustrates the three distinct groups defined by the WHO, namely the VOC (Delta), the VOI (Eta), and the VUM (20B). The Delta variant, which appeared slightly more predominant than the others, had more mutations in the majority of isolates (61.1%). This Delta variant has been shown to be highly transmissible and has spread worldwide among fully vaccinated and unvaccinated individuals [51]. Indeed, among the five COVID-19 epidemic waves that Mauritania has experienced, the third one, which was mainly due to the Delta variant, was the deadliest wave with a total of 517 deaths, representing 52% of the total number of deaths (i.e., 997) attributable to COVID-19 in Mauritania during the pandemic. 

An interesting finding of this study was that 61.1% of SARS-CoV-2 isolates were retrieved from individuals who had received different vaccines (i.e., 25.9% after receiving the first dose of AstraZeneca, 31.5% ‘protected’ with the Sinopharm vaccine, and 3.7% after vaccination with the Johnson & Johnson vaccine). This finding suggests that vaccination against COVID-19 could not fully prevent SARS-CoV-2 infection. However, due to insufficient clinical and hospitalization data on patients included in the present study, it cannot be deduced from our results whether vaccinated patients had milder infections than in unvaccinated patients. Nonetheless, a recent meta-analysis that assessed the efficacy of various COVID-19 vaccines in preventing SARS-CoV-2 infections of different severities showed that the efficacy of COVID-19 vaccines is higher to prevent severe infection and death than to prevent a milder infection and that vaccine efficacy wanes over time but can be enhanced by a booster [52].

As elsewhere in the world, Mauritania had to face important challenges during the COVID-19 pandemic. One of them was the capacity to accurately diagnose SARS-CoV-2 infections and rapidly characterize the newly emerging variants. Therefore, implementation of the NGS techniques during the COVID-19 epidemic has been of great interest in detecting and assessing the protective level of a particular vaccine [53] and, most importantly, in guiding the countries in making rapid and informed public health decisions.

Our study has several limitations. SARS-CoV-2 isolates were collected over only three months. It is possible that, during the study period, other variants were present but not sampled. Therefore, we are aware that the present finding may document only part of the SARS-CoV-2 genetic diversity during the study period. Moreover, our study was focused on, and limited essentially to, the analysis of SARS-CoV-2 genome. The patients’ full hospital records were not available for consultation, precluding us from analyzing our sequence data in the light of clinical presentation, co-morbidity, treatment, outcome, and short- and long-term complications. In addition, the total number of samples sequenced and analyzed in the present study was relatively small. The limited number of sequences did not allow for a more robust statistical analysis of a possible relationship between the subsets of different anti-COVID-19 vaccines and different clades and variants of SARS-CoV-2 found in Mauritania. Nonetheless, the present study is one of the few studies that have characterized the whole genomes of Mauritanian SARS-CoV-2 isolates by NGS. Further monitoring and analysis of a higher number of viral samples will be required as the current pandemic seems to have taken hold in the world and may even have stabilized [3]. 

## 5. Conclusions

Several variants of SARS-CoV-2 were present in Mauritania between September and November 2021. As Mauritania, like most West African countries, is resource limited to undertake viral genome sequencing, especially by NGS, an establishment of a mutualized sub-regional sequencing platform will be necessary to ensure continuous monitoring of SARS-CoV-2 variants as an essential step to track the potential reduction in COVID-19 vaccine efficacy, increased viral transmissibility, and disease severity.

## Figures and Tables

**Table 1:** Genotypes identified among SARS-CoV-2 clinical isolates during the COVID-19 epidemic in Mauritania (September–November 2021).

WHO Classification *	Clade	Pango Lineage	WHO Label	Origin	Frequency (%)
VOC	21J	AY.34 (alias B.1.617.2.34)	Delta	Multiple countries	31 (57.4)
	21I	AY.61 (alias B.1.617.2.61)	Delta	Italy	1 (1.9)
		AY.75 (alias B.1.617.2.75)	Delta	USA and Europe	1 (1.9)
	20I	B.1.1.7	Alpha	United Kingdom	1 (1.9)
	20B	B.1.1.528	20B	South Africa	1 (1.9)
	20A	B.1	20A	Italy	1 (1.9)
VOI	21D	B.1.525	Eta	Nigeria	11 (20.4)
	20B	AZ.3	20B	USA	1 (1.9)
	20B	B.1.1.318	20B	Multiple countries	3 (5.5)
VUM	20A	B.1.620	20A	Potentially from Cameroon	3 (5.5)

* The WHO classification is based on the 2023 updated definitions of SARS-CoV-2 variants established by the World Health Organization (WHO) [36]. Pango, Phylogenetic Assignment of Named Global Outbreak; VOC, variant of concern; VOI, variant of interest; VUM, variant under monitoring.

**Table 2:** Relationship between virus variants or clades and the vaccination status of the Mauritanian patients.

Variant or Clade	Vaccinated	Unvaccinated
VOC	21	15
VOI + VUM	12	6
Total	33	21
Clade AY.34	18	13
Other clades	15	8
Total	33	21

VOC, variants of concern; VOI, variants of interest; VUM, variants under monitoring. VOC included 33 Delta, one Alpha, one 20A, and one 20B (B.1.1.528) variants. VOI included 11 Eta and four 20B variants. VUM included three 20A variants. See Table 1 for details. The association between the variants (VOC versus VOI and VUM pooled together) or clades (Clade AY.34 versus five other clades) and the vaccination status of the patients was not statistically significant (two-tailed *p*-values, 0.7678 and 0.7784, respectively, using Fisher’s exact test).

**Table 3:** Notable mutations in the spike protein of both Delta and Eta variants among SARS-CoV-2 clinical isolates during the COVID-19 epidemic in Mauritania (September-November 2021).

Variant *	AA ** Substitution	No. Isolates (%)	Reported Effect of the Mutation	Reference
Delta (*n = 33*)	T19R	32 (97)	Improved transmissibility Reduced neutralization efficacy of RBD-directed monoclonal antibodies Escape from (HLA)-24-restricted cellular immunity Reduction in neutralization efficacy of vaccines and convalescent sera	[37,38]
	L452R	30 (91)		
	T478K	32 (97)		
	D614G	33 (100)		
	P681R	33 (100)		
	D950N	33 (100)		
Eta (*n = 9*)	Q52R	9 (100)	Potentially reduced vaccine efficacy Potentially reduced neutralization by vaccine sera Enhanced binding to hACE2 receptor	[39,40]
	E484K	8 (88.9)		
	D614G	9 (100)		
	Q677H	9(100)		
	F888L	8 (88.9)		

* Delta variant was represented by three sublineages, namely AY.34 (alias B.1.617.2.34), AY.61 (alias B.1.617.2.61), and AY.75 (alias B.1.617.2.75), while Eta variant was represented by only B.1.525 sublineage. ** AA, amino acid; hACE2, human angiotension-converting enzyme 2; HLA, human leukocyte antigen; RBD, receptor-binding domain.

## Data Availability

Genomic sequence data of the Mauritanian isolates of SARS-CoV-2 characterized and presented in this study are available in GenBank (accession numbers OR353179–OR353230).

## Supplementary Materials

The following supporting information can be downloaded at https://www.mdpi.com/article/10.3390/genes15030361/s1, Table S1: Genomic features of Mauritanian clinical isolates of SARS-CoV-2: mutations, amino acid substitutions and deletions, coverage, clade, and Pangolin lineage.

## Abbreviations

AA, amino acid; COVID-19, coronavirus disease 2019; CPP, COVIDSeq primer pool; Ct, cycle threshold; hACE2, human angiotension-converting enzyme 2; HLA, human leukocyte antigen; IHU-MI, Institut Hospitalo-Universitaire–Méditerranée Infection; INRSP, Institut National de Recherche en Santé Publique (National Institute of Public Health Research); NGS, next-generation sequencing; SARS-CoV-2, severe acute respiratory syndrome coronavirus 2; RBD, receptor-binding domain; RT-PCR, reverse transcription-polymerase chain reaction; SD, standard deviation; S glycoprotein, spike glycoprotein; VOC, variants of concern; VOI, variants of interest; VUM, variants under monitoring; WHO, World Health Organization.
